# Co-Administration of Influenza and COVID-19 Vaccines: A Cross-Sectional Survey of Canadian Adults’ Knowledge, Attitudes, and Beliefs

**DOI:** 10.3390/pharmacy12020070

**Published:** 2024-04-17

**Authors:** Sherilyn K. D. Houle, Ajit Johal, Paul Roumeliotis, Bertrand Roy, Wendy Boivin

**Affiliations:** 1 School of Pharmacy, University of Waterloo, Waterloo, ON N2L 3G1, Canada; 2 Travelrx and Immunize.io, Vancouver, BC V5Z 3Y1, Canada; ajit@immunize.io; 3 School of Epidemiology and Public Health, University of Ottawa, Ottawa, ON K1N 6N5, Canada; drpaul@drpaul.com; 4 CSL Seqirus/Medical Affairs Americas, 16766 TransCanada, Suite 504, Kirkland, QC H9H 4M7, Canada; bertrand.roy@seqirus.com

**Keywords:** influenza, influenza vaccines, pharmaceutical services, COVID-19, COVID-19 vaccines

## Abstract

Vaccination rates against both influenza and COVID-19 fall short of targets, especially among persons at risk of influenza complications. To gain insights into strategies to boost influenza vaccine coverage, we surveyed 3000 Canadian residents aged ≥ 18 years and examined their knowledge and receipt of co-administered influenza and COVID-19 vaccines. During the 2022–2023 influenza season, 70% of respondents reported being aware the influenza and COVID-19 vaccines could be co-administered, but only 26.2% (95% CI, 23.6% to 28.8%) of respondents received them together. The most common reason for not getting the vaccines together was receipt of the COVID-19 vaccine before the annual influenza vaccine was available (reported by 34.5% [31.2% to 37.7%]). Lack of interest in co-administration was reported by 22.6% (20.8% to 24.3%); of this group, 20.8% (17.1% to 24.5%) reported seeing no benefit in receiving the two vaccines together and 17.2% (13.5% to 20.9%) were concerned about compounded adverse effects from the two vaccines. These results support the willingness of most Canadians to receive COVID-19 and influenza vaccines at the same time. Co-administration is a viable strategy to improve uptake of influenza vaccines, especially if health professionals proactively offer education and co-administration of influenza and COVID-19 (or other) vaccines as appropriate to clinical need.

## 1. Introduction

Influenza is currently the tenth and coronavirus disease 2019 (COVID-19) the fourth leading cause of death in the Canadian general population [1]. These diseases also cause tremendous morbidity and significant economic impacts [2,3,4,5,6,7]. Each year in Canada, an average of 12,000 hospitalizations and 3500 deaths are attributed to influenza, while over 58,000 Canadians have died from COVID-19 since the start of the pandemic through the end of the year 2023 [3,7]. The direct medical costs for each hospitalization amount to an average of $14,612 CAD, and lost productivity and premature death due to influenza place a high burden on society [2,4]. Another indirect consequence of both influenza and COVID-19 is an increased risk of cardiovascular events such as myocardial infarctions and strokes. The economic burden on individuals and society of these sequelae is also very high [8,9,10].

To reduce these burdens, the National Advisory Committee on Immunization (NACI) and Public Health Agency of Canada (PHAC) recommend immunization with influenza and COVID-19 vaccines. COVID-19 vaccination rates exceed 80% for both a single dose and completion of the primary series of vaccines against SARS-CoV2, although only 29%, 26%, and 13% of Canadians report receiving one, two, and three booster doses, respectively [11,12]. For influenza, PHAC has set a target of 80% coverage for high-risk persons by 2025. This group includes adults ≥ 65 years, those aged 18–64 with NACI-defined chronic medical conditions (including hypertension, diabetes, chronic lung disease, heart disease, body mass index [BMI] ≥ 40 kg/m^2^, anemia, immune disorder, and cancer, among other conditions), and healthcare workers who may come in contact with these groups [13]. Rates of influenza vaccination among adults aged ≥ 65 years have remained stable since before the COVID-19 pandemic (ranging from 70% to 74% between 2019 and 2023). Yet coverage among adults < 65 years with chronic conditions was only 43% in the 2022–2023 influenza season—well short of the PHAC target—and even decreased during the pandemic [12,14].

NACI considers co-administration of COVID-19 and influenza a safe strategy for reducing barriers to vaccine coverage [13,15]. Before 2019, influenza vaccination rates were rising slowly but steadily [16], and restoring these trends is essential to Canadian public health, especially for those at high risk. Here, we report on rates of and interest in co-administration of influenza and COVID-19 vaccines from a cross-sectional, self-reported, online survey of 3000 Canadians aged ≥ 18 years between 5 and 21 December 2022. 

## 2. Materials and Methods

The study design and survey findings regarding vaccination in a pharmacy setting and vaccine knowledge and uptake among high-risk populations have been previously published [17,18]. We collected self-reported data on Canadians’ knowledge, attitudes, and beliefs about influenza and COVID-19 vaccines using a structured questionnaire available on Internet-accessible platforms in both English and French (Supplementary Materials File S1). Survey participants were randomly recruited from the Léger Opinion (LEO) consumer panel and included a prespecified high-risk subgroup comprising persons meeting at least one NACI-defined high-risk criterion [13]. The survey sample was representative of the Canadian population, with recruitment quotas by age, gender, and region set based on 2021 Canadian census data [19]. During the recruitment phase, sampling was adjusted to ensure all age, gender, and regional cohorts were filled in proportions representative of Canadian residents. At the end of data collection, to further ensure representativeness, data were weighted by age, region, and gender based on 2021 census data [19].

All survey participants provided informed consent and their information was fully anonymized before data collection, aggregation, and analysis. The Veritas Independent Review Board (IRB) approved the study design.

The study questionnaire (Supplementary Materials File S1), which was accessible via computer, smartphone, or tablet on the Decipher Survey Platform (Forsta, Vancouver, BC, Canada), consisted of ~70 questions on demographics, vaccination status, general attitudes toward vaccines, interactions with healthcare providers, and knowledge and awareness of different types of influenza vaccines. Survey completion took ~15 min. Most questions were multiple choice, but some required respondents to fill in a blank answer box. These respondent-supplied answers were grouped into relevant categories in an iterative coding process.

For analysis, survey respondents were divided into subgroups based on age (<35, 35–64, and ≥65 years), risk of influenza complications (high vs. not high risk) based on NACI high-risk criteria, and history of vaccination with influenza or COVID-19 vaccines (previously vaccinated or not previously vaccinated). Data were analyzed using coding software (Coder, Ascribe, Cincinnati, OH, USA), and statistical analyses were performed using Q/SPSS and Microsoft Excel. Additional details on data collection and statistical analysis have been previously published [17,18].

## 3. Results

As previously reported, the final survey population of 3000 had proportional representation of the Canadian populace in terms of gender, racial and ethnic groups, and geography. Of all respondents, 50.5% (95% CI, 48.7% to 52.3%) met at least one NACI high-risk criterion, with 37.2% (35.4% to 39.0%) high-risk due to an underlying medical condition. Most respondents (72.6% [70.8% to 74.4%]) had been vaccinated against influenza at some point in their lives, but less than half had received an influenza vaccine during the 2021–2022 (47.7% [45.9% to 49.5%]) and 2022–2023 (47.3% [45.5% to 49.1%]) influenza seasons [17,18]. Among older adults aged ≥ 65 years, rates of influenza vaccination were high; 75.7% (95% CI, 72.0% to 79.5%) received an influenza vaccine in the 2021–2022 influenza season, followed by 77.4% (95% CI, 73.7% to 81.1%) in the 2022–2023 season. However, among people aged 18–64 years with medical conditions putting them at risk of influenza complications, only 49.2% (95% CI, 45.5% to 52.8%) and 46.4% (95% CI, 42.5% to 50.1%) had received an influenza vaccine in the 2021–2022 and 2022–2023 influenza seasons, respectively. In this subgroup, influenza vaccination rates were somewhat higher (>64%) among those who were aware of their high-risk status. The most commonly reported reason for not being vaccinated among high-risk respondents was “I did not get around to it”; <20% of respondents who were not vaccinated in the 2022–2023 season expressed vaccine hesitancy due to concerns with the influenza vaccine or its side effects or efficacy [17].

Receipt of at least one dose of a COVID-19 vaccine since the start of the pandemic was reported by 93.2% (95% CI, 91.4% to 95.0%) of respondents overall, including 93.6% (89.9% to 97.3%) of respondents aged 18–64 years with a high-risk medical condition and 96.8% (93.1% to 100%) of respondents aged ≥ 65 years. During the 2022–2023 season, 26.2% (23.6% to 28.8%) of respondents reported receiving a COVID-19 and an influenza vaccine at the same time. Co-administration rates were generally lower among high-risk subgroups (age ≥ 65 years, 23.9% [19.7% to 28.1%] and respondents aged 18–64 years with ≥1 high-risk medical condition (25.4% [19.0% to 30.9%]) than in persons aged 18–64 without high-risk medical conditions (28.4% [24.2% to 32.7%]) [17].

**Figure 1:**
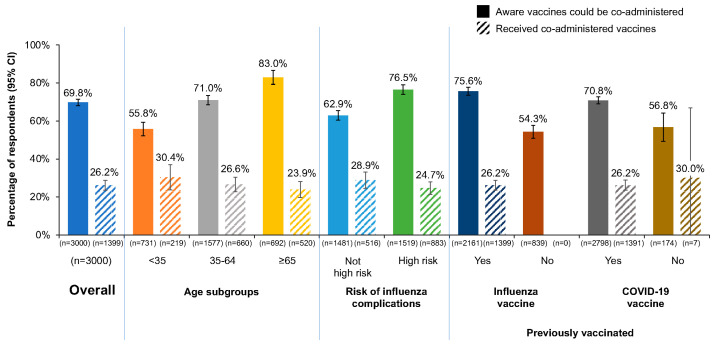
Awareness (solid columns) and receipt (hatched columns) of influenza and COVID-19 vaccine co-administration overall and among age, high risk, and previously vaccinated subgroups. Rates of awareness determined by respondents answering “yes” to “Before today, were you aware that healthcare providers can administer the flu vaccine at the same time (or any time before or after) administering the COVID-19 vaccine?” (QE13; see Supplementary Materials File S1). CI, confidence interval.

Although less than one-third of respondents reported receiving an influenza and COVID-19 vaccine together during the 2022–2023 influenza season, 69.8% (95% CI, 68.0% to 71.5%) reported being aware the vaccines could be co-administered (Figure 1). Awareness was higher among high-risk respondents than those not at high risk, among respondents aged ≥ 65 years than younger respondents, and among those who had previously received an influenza or COVID-19 vaccine than among the unvaccinated.

**Figure 2:**
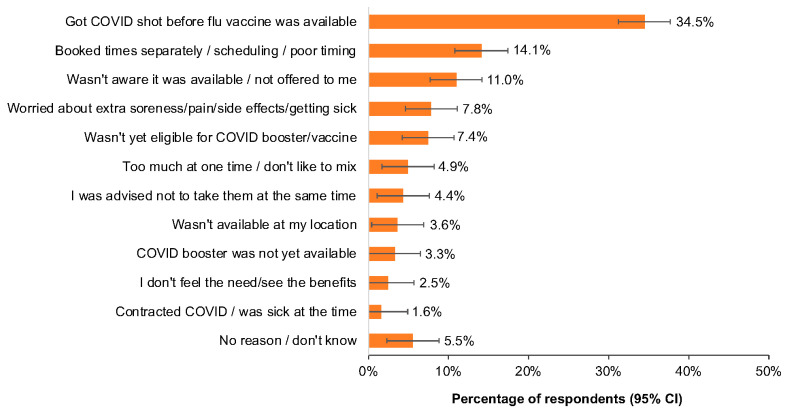
Responses to “Why did you not receive influenza vaccination at the same time as the COVID-19 booster this flu season?” (QE17, see Supplementary Materials File S1). CI, confidence interval.

As shown in Figure 2, the most common reasons given for not receiving co-administration were receipt of a COVID-19 vaccine before the annual influenza vaccine was available (34.5% [95% CI, 31.2% to 37.7%]) or a scheduling conflict (14.1% [95% CI, 10.8% to 17.4%]); 4.4% (95% CI, 1.1% to 7.6%) of respondents reported being told not to take the vaccines together, and 11.0% (95% CI, 7.7% to 14.2%) reported that they were not aware that co-administration was available. Less than 10% reported worry about “extra” adverse effects such as soreness or illness (7.8% [95% CI, 4.6% to 11.1%]), a belief that the two vaccines together would be “too much” (4.9% [95% CI, 1.7% to 8.2%]), or that co-administration offered no benefits (2.5% [95% CI, 0% to 5.7%]).

**Figure 3:**
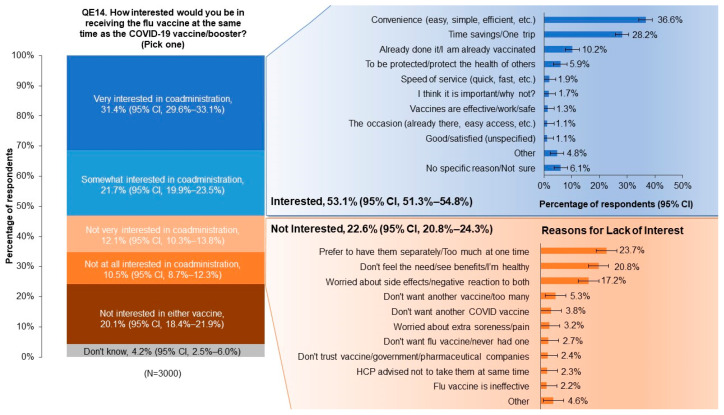
Respondents’ reported levels of interest in receiving influenza and COVID-19 vaccines together ((**left**); QE14; see Supplementary Materials File S1) and reasons for or against interest ((**right**); QE15; see Supplementary Materials File S1). Open answer refers to respondent-supplied answers (in which they filled in a blank box), which were grouped into relevant categories shown in the (**right**) panel. CI, confidence interval.

Of the total population, 53.1% (95% CI, 51.3% to 54.8%) expressed interest in co-administration of the influenza and COVID-19 vaccines (Figure 3). In this group, the most commonly reported reasons for interest were associated with convenience (36.6% [95% CI, 34.2% to 39.1%]) and time savings (28.2% [95% CI, 25.7% to 30.6%]). Of the 22.6% (95% CI, 20.8% to 24.3%) of respondents who were not interested in co-administration, 23.7% (95% CI, 20.0% to 27.4%) expressed a preference for receiving the vaccines separately, 20.8% (95% CI, 17.1% to 24.5%) reported seeing no benefit in receiving the two vaccines together, and 17.2% (95% CI, 13.5% to 20.9%) were concerned about adverse effects.

## 4. Discussion

In this analysis of data from a cross-sectional, self-reported, online survey of 3000 Canadian adults ≥ 18 years conducted between 5 and 21 December 2022, we found generally high acceptance of influenza and COVID-19 vaccines as determined by a majority of respondents having received an influenza and/or a COVID-19 vaccine in the past. Most survey respondents (69.8%) were aware that the influenza and COVID-19 vaccines could be administered at the same time, and 53.1% of respondents expressed interest in co-administration as a convenient and/or time-saving strategy. However, only 26.2% of respondents received the two vaccines together. Of those who received the vaccines separately, 34.5% were vaccinated against COVID-19 before the annual influenza vaccine was available, and 22.6% were not interested in co-administration. Out of this group, 20.8% reported seeing no benefit in receiving the two vaccines together and 17.2% were concerned that adverse effects from the two vaccines might be compounded.

Our findings are consistent with results from the latest PHAC seasonal influenza vaccination survey, in which 30% of adults had received a COVID-19 vaccine at the same time as an influenza vaccine (26.2% in our survey), and 66% indicated that receipt of a COVID-19 vaccine would not affect their likelihood of receiving an influenza shot. In addition, 53% of PHAC respondents cited convenience as a major reason for receiving the COVID-19 and influenza vaccinations together [12]. In our survey, ~65% of respondents gave similar reasons for being interested in co-administration. Compared to our findings, a higher proportion of PHAC survey respondents (42% vs. ≤17% in our study) reported potentially increased adverse events as a deterrent to receiving vaccine co-administration [12]. Within the high-risk group, respondents to our survey expressed vaccine hesitancy due to concerns about the influenza vaccine or its side effects (13.5%) or efficacy (3.2%) of influenza vaccines. Among high-risk respondents, 21% gave no specific reason for not receiving an influenza vaccine, and 15.3% said they “did not get around to it” [17]. This finding is generally consistent with other recent surveys among Canadians, even as vaccine hesitancy remains high in other populations [14,20,21,22,23]. 

Motivating Canadians—especially those at high risk from respiratory infections—to prioritize influenza and COVID-19 vaccinations for themselves and their family members is an important goal for public health authorities, as vaccination rates in Canada remain well below PHAC-recommended targets of 80% coverage for high-risk persons [14]. Among adults aged 18–64 years with high-risk medical conditions, coverage rates in recent influenza seasons were 44% in 2019–2020, before the COVID-19 pandemic, dropping to a low of 38% in 2021–2022 during the pandemic, and rising back to 43% in 2022–2023 [12,14]. Meanwhile, in terms of COVID-19 vaccine coverage, 84% of Canadians received at least one dose of a COVID-19 vaccine and 68% received at least one booster dose, but only 15% are fully vaccinated per PHAC recommendations [11,12]. It is possible that increased education on the importance of vaccination may increase rates of vaccination against both influenza and COVID-19. In the Canadian Community Health Survey (CCHS) study conducted between 2009 and 2018, Canadians with cardiovascular disease were twice as likely to be vaccinated against influenza if they had a healthcare provider than if they did not [21].

All healthcare professionals can play a role in addressing concerns about lack of benefit or increased adverse events from co-administration. However, results from our survey point to a communication gap between healthcare providers and their patients. As previously reported, ≤51% of high-risk respondents to our survey (43% of adults aged 18–64 years with medical conditions and 51% of older adults aged ≥ 65 years) had spoken with a healthcare provider about influenza vaccines during the previous season [17], and in this analysis, only 39.1% of all respondents had spoken with a healthcare provider about co-administration of the influenza and COVID-19 vaccines during the 2022–2023 season. By proactively providing information about influenza and COVID-19 vaccines and co-administration, healthcare providers could help increase overall uptake of both vaccines, especially among high-risk Canadians. For example, the use of presumptive statements has been found to significantly improve vaccine uptake [24] and could be applied to co-administration (e.g., “You are due for your flu and COVID-19 shots. Let us take care of that now.”). In addition, the Canadian Influenza Immunization Awareness Campaign offers tools that could help bridge this gap, including educational resources on influenza that target at-risk groups [25].

Disseminating information about convenient ways to obtain vaccines may also increase uptake. In Canada, vaccines may be obtained from pharmacies, physicians’ offices, public health offices, workplace clinics, and other sites. Pharmacies now provide the majority of influenza vaccines in Canada. According to the PHAC, 52% of Canadians received the influenza vaccine at a pharmacy during the 2022–2023 season [12]. We found a similar rate in our survey, with 54% of respondents reporting a pharmacy-administered influenza vaccine during the same season and 94% of those individuals reporting high satisfaction with the experience [18]. Pharmacists, along with all other healthcare providers, can play a key role in educating Canadians about the importance of vaccines for respiratory diseases and providing convenient access to vaccines. These strategies will increase in importance as more vaccines for respiratory conditions become available, including new formulations of COVID-19 vaccines and the vaccine against respiratory syncytial virus (RSV).

## 5. Conclusions

Our survey results support the willingness of most Canadians to receive COVID-19 and influenza vaccines at the same time. Uptake of COVID-19 boosters has been decreasing, and influenza vaccination coverage lags behind PHAC targets, especially in adults younger than 65 years of age with chronic medical conditions. As new COVID-19 boosters, as well as vaccines against RSV, become available, co-administration including with influenza vaccine will be an increasingly important strategy to support uptake of all indicated vaccines.

## Data Availability

Data available from the authors upon request.

## Supplementary Materials

The following supporting information can be downloaded at: https://www.mdpi.com/article/10.3390/pharmacy12020070/s1, File S1: online resource 1: survey instrument.
