# Supplementary material for: Co-Administration of Influenza and COVID-19 Vaccines: A Cross-Sectional Survey of Canadian Adults’ Knowledge, Attitudes, and Beliefs
Source: Pharmacy (Basel). 2024 Apr 17;12(2):70. doi: 10.3390/pharmacy12020070 (PMC11054434; doi:10.3390/pharmacy12020070)
Supplement: Supplementary file 1 [file pharmacy-12-00070-s001.zip › pharmacy-2890513-supplementary.pdf]

SUPPLEMENTARY MATERIALS FILE S1
ONLINE RESOURCE 1. SURVEY INSTRUMENT FOR:

Co-administration of Influenza and COVID-19 Vaccines: A Cross-Sectional Survey of Canadian Adults' Knowledge, Attitude, and Beliefs

Sherilyn K.D. Houle, BSP, PhD; Ajit Johal, BSc (Pharm), BCPP, RPh; Paul Roumeliotis, MD, MPH, FRCP(C); Bertrand Roy, PhD; Wendy Boivin, PhD

Corresponding author:
Wendy Boivin, PhD
Seqirus / Medical Affairs Americas
16766 TransCanada, Suite 504, Kirkland, QC, H9H 4M7, Canada
Email: Wendy.Boivin@Seqirus.com

Published in *MDPI Pharmacy*

Thank you for agreeing to participate in our survey. Please rest assured that your answers will remain confidential. The survey will take approximately 15 minutes to complete.

SCREENER

S1. Please provide your age.

_____ years
Prefer not to say **[TERMINATE]**

PN: TERMINATE IF THE RESPONDENT IS BELOW 18 YEARS OF AGE

S2. In which province or territory do you live?

British Columbia	1
Alberta	2
Saskatchewan	3
Manitoba	4
Ontario	5
Quebec	6
New Brunswick	7
Nova Scotia	8
Prince Edward Island	9
Newfoundland	10
Nunavut	11
Northwest Territories	12
Yukon	13

S3. What is your gender?

Male	1
Female	2
Non-binary	3
Other (specify): ______________	96
Prefer not to say	99

MAIN QUESTIONNAIRE

SECTION A: HEALTHCARE USE

A1. How many times have you consulted the following health care providers for any condition, either in-person or virtually via telemedicine, in the last 12 months?

General Practitioner / Family Physician	___ # **[PN: RANGE 0-50]**
Another physician	___ # **[PN: RANGE 0-50]**
Pharmacist	___ # **[PN: RANGE 0-50]**
Nurse	___ # **[PN: RANGE 0-50]**
Don’t know/Not sure	98
Prefer not to say	99

SECTION B: VACCINATION IN GENERAL

B1. Please indicate the extent to which do you agree or disagree with the following statements about vaccines in general.

		Strongly disagree	Somewhat disagree	Somewhat agree	Strongly agree	Don’t know/Not sure	Prefer not to say
1	In general, I consider vaccines to be important for my health	1	2	3	4	98	99
2	I know enough about vaccines to make an informed decision about getting vaccinated	1	2	3	4	98	99
3	If my doctor suggests a vaccination, I would get vaccinated without hesitation	1	2	3	4	98	99
4	If a pharmacist suggests a vaccination, I would get vaccinated without hesitation	1	2	3	4	98	99

B2. Please indicate the extent to which do you agree or disagree with the following statements.

		Strongly disagree	Somewhat disagree	Somewhat agree	Strongly agree	Don’t know/Not sure	Prefer not to say
1	Since the pandemic, there is less focus on vaccines other than COVID-19 vaccine	1	2	3	4	98	99
2	Since the pandemic, there is less focus on the flu vaccine than the COVID vaccine	1	2	3	4	98	99

SECTION C: COVID-19 VACCINATION

C1. Have you been vaccinated against COVID-19?

Yes, one dose	1
Yes, two doses	2
Yes, three doses or more	3
No, but intend to	4
No, and do not intend to	5
Prefer not to say	98

SECTION D: DISCUSSING FLU VACCINES WITH HCPs

We would now like to ask you some questions about the flu vaccine, your vaccination status and how you feel about the process.

D1. Did you discuss flu vaccines with a health care provider (e.g., physician, nurse, pharmacist, etc.) this flu season (that is, between September and now)?

Yes, more than once	1
Yes, once	2
No	3
Can't remember	99

PN: ASK IF YES AT D1

D2. Which health care provider do / did you discuss flu vaccines with? *Please select all that apply.*

General Practitioner / Family Physician	1
Another physician	2
Pharmacist	3
Nurse	4
Other – please specify ______	96

PN: ASK FOR ALL HCPs SELECTED AT D2

D3. As far as you recall, what did you discuss about flu vaccines with the health care provider?

PN: ASK IF YES AT D1 AND FOR ALL HCPs SELECTED AT D2

D4. How satisfied were you with the discussion with **[INSERT D2 RESPONSES]** about the flu vaccines?

Not at all satisfied<br>1	Not very satisfied<br>2	Neither satisfied nor dissatisfied<br>3	Somewhat satisfied<br>4	Very satisfied<br>5

D5. Why do you say you are **[INSERT D4 RESPONSE]** with the discussion you had with **[INSERT D2 RESPONSES]** about the flu vaccines?

SECTION E: FLU VACCINATION

The next few questions will be around flu vaccination status…

E1. Have you ever received the flu vaccine?

Yes	1
No	2

PN: ASK IF CODE 1 SELECTED AT E1

E2. Did you receive the flu vaccine this flu season (that is, between September and now)? And how about last flu season (that is, between September 2021 and March 2022)?

	**This Flu Season** (between September and now)	**Last Flu Season** (between September 2021 and March 2022)
Yes	1	1
No	2	2

PN: ASK IF CODE 1 SELECTED AT E1

E3. Have you ever received the flu vaccine in previous years (that is, in the flu season of 2020 or before)?

Yes	1
No	2

PN: ASK IF CODE 1 SELECTED AT E2

E4a. Where did you receive your flu vaccine? (Select only one)
PN: FILTER COLUMN BASED ON E2. ASK ONLY IF YES SELECTED AT E2

	**This Flu Season** (between September and now)	**Last Flu Season** (between September 2021 and March 2022)
Place of employment	1	1
Pharmacy	2	2
Physician's office or medical clinic	3	3
Hospital	4	4
Community-based public health clinic, such as in a shopping mall or library / CLSC **[ONLY FOR QC]**	5	5
Public Health Department	6	6
Other (Specify)	96	96
Can't remember	98	98
Prefer not to say	99	99

PN: ASK IF SELECTED DIFFERENT OPTIONS FOR BOTH COLUMNS AT E4a

E4b. You mentioned you received your flu vaccine this flu season (between September and now) at _____ **[INSERT E4A COLUMN 1 RESPONSE]** while last flu season (that is, between September 2021 and March 2022) you received it at _____ **[INSERT E4A COLUMN 2 RESPONSE].** Is there a reason you changed your place of vaccination?

PN: ASK IF CODE 1 SELECTED AT E3 BUT NOT AT E2

E4c. As far as you recall, where did you receive your last flu vaccine? (Select only one)

Place of employment	1
Pharmacy	2
Physician's office or medical clinic	3
Hospital	4
Community-based public health clinic, such as in a shopping mall or library / CLSC **[ONLY FOR QC]**	5
Public Health Department	6
Other (Specify)	96
Can't remember	98
Prefer not to say	99

PN: ASK IF CODE 1 SELECTED AT E1

E5. Where would you prefer to get a flu vaccine? Please rank top 3.

Place of employment	1
Pharmacy	2
Physician's office or medical clinic	3
Hospital	4
Community-based public health clinic, such as in a shopping mall or library / CLSC **[ONLY FOR QC]**	5
Public Health Department	6
Other (Specify)	96
Can't remember	98
Prefer not to say	99

PN: ASK IF CODE 1 SELECTED AT E1

E6. You mentioned you got your last flu vaccine at **[INSERT E4a RESPONSE]**. Did you encounter any of the following difficulties in scheduling an appointment for getting the flu vaccine? (Select all that apply)

PN: RANDOMIZE

Limited appointment availability	1
Transportation to get the appointment was a problem	2
I didn't know who to call to schedule an appointment	3
Concern about being exposed to COVID-19	4
No one could take care of my spouse/partner, children or other loved ones during the appointment	5
Lack of walk-in options	6
The vaccine was not offered at my usual/a convenient location	7
Other (specify): _______	96
I didn't encounter any difficulties in scheduling an appointment **[MUTUALLY EXCLUSIVE]**	97
I did not take any action to get vaccinated this flu season **[MUTUALLY EXCLUSIVE]**	98
Don't know/Not sure **[MUTUALLY EXCLUSIVE]**	99
Prefer not to answer	99

PN: ASK IF CODE 1 SELECTED AT E1

E7. Thinking about the last time you got a flu vaccine, what are the reasons you decided to receive the flu vaccine? (Select all that apply). **PN: RANDOMIZE**

I am worried about getting both the flu and COVID-19	1
I want to prevent infection / I do not want to get sick	2
I am at risk because of my health condition	3
I am at risk because of my age	4
I receive it every year / It's just something I've always done	5
To protect the health of others	6
Recommendation from my family physician	7
Recommendation from another physician	8
Recommendation from a pharmacist	9
It was encouraged by family members, colleagues or friends	10
It's free	11
Concerned about having to go to hospital if I get flu, when they are overcrowded	12
Other (specify): _____________________	96
Don't know/Not sure	98
Prefer not to say	99

PN: ASK IF CODE 2 SELECTED AT E2 FOR LAST FLU SEASON

E8. What was the *most important* reason why you did not receive the flu vaccine this flu season (that is, between September and now)?

PN: RANDOMIZE

I don't believe in vaccines	1
Flu vaccines don't work	2
I am healthy, and/or never get the flu	3
I am healthy, not at risk of influenza-related complications or hospitalization	4
Getting the flu doesn't make me that sick	5
I did not get around to it	6
I have concerns about the flu vaccine, and/or its side effects	7
I have concerns about being exposed to COVID-19 while getting the flu vaccine	8
I got the flu before I had the opportunity to get the flu vaccine	9
It was too expensive (cost of the vaccine) **[ASK QC ONLY]**	10
I was not able to get an appointment	11
No specific reason, I just didn't get it	12
I received COVID-19 vaccine so I did not feel a need to receive the flu vaccine because COVID is more severe than flu	13
Only wanted to get one vaccine this fall	14
Concerned about getting both the flu & COVID vaccines close together	15
Other (specify): ____	96
Don't know/Not sure	98
Prefer not to say	99

E9. To what extent do the following factors impact your decision whether to get a flu vaccine or not? *Please use a scale from 1-5, where 1 is 'does not impact at all' and 5 is 'has a huge impact'.*

PN: RANDOMIZE

Does not impact at all 1	2	3	4	Has a huge impact 5	Don't know/ Not sure 98

Available at a nearby pharmacy	1
Available at my family doctor's clinic	2
Ease of booking an appointment	3
Convenient location	4
Easy transportation to get to the location	5
Faster / less time consuming to get the vaccine	6
Walk-in appointment available	7
Recommendation from my family physician	8
Recommendation from another physician	9
Recommendation from a pharmacist	10
Recommendation from a friend / relative	11
Whether I have had any other vaccinations near the date	12
How prevalent the flu usually is in my community	13
How full the hospitals are	
Other – please specify_____	96

PN: ASK IF RECEIVED COVID-19 VACCINE (C1=1, 2 and 3)

E10a. Did receiving a COVID-19 vaccine make you more or less likely to get the flu vaccine this flu season (that is, between September and now)?

Receiving a COVID-19 vaccine made me **much less likely** to get a flu vaccine	1
Receiving a COVID-19 vaccine made me **somewhat less likely** to get a flu vaccine	2
Receiving a COVID-19 vaccine had **no change in likelihood** to get a flu vaccine	3
Receiving a COVID-19 vaccine made me **somewhat more likely** to get a flu vaccine	4
Receiving a COVID-19 vaccine made me **much more likely** to get a flu vaccine	5
Don't know/Not sure	98
Prefer not to say	99

PN: ASK IF CODE 1, 2, 4 OR 5 AT E10

E10b. Please briefly explain <u>why</u> receiving a COVID-19 vaccine influenced your decision to receive the flu vaccine this flu season (that is, between September and now).

PN: ASK IF NOT RECEIVED FLU VACCINE LAST FLU SEASON (E2 =2, LAST FLU SEASON COLUMN)

E11a. Do you intend to receive the flu vaccine during the 2022-23 flu season (i.e., between fall 2022 and spring 2023)?

Yes, definitely	1
Yes, probably	2
Probably not	3
Definitely not	4
Don't know/Not sure	98
Prefer not to say	99

E11b. Do you intend to receive the flu vaccine next flu season (that is, between September 2023 and March 2024)?

Yes, definitely	1
Yes, probably	2
Probably not	3
Definitely not	4
Don't know/Not sure	98
Prefer not to say	99

PN: ASK IF CODE 1 OR 2 SELECTED AT E11b

E12. Please briefly explain <u>why</u> you **[PN: INSERT E11b RESPONSE]** intend to get the flu vaccine next flu season (that is, between September 2023 and March 2024).

E13. Before today, were you aware that healthcare providers can administer the flu vaccine at the same time (or anytime before or after) administering the COVID-19 vaccine?

Yes	1
No	2

E14. Knowing this, how interested would you be in receiving the flu vaccine at the same time as the COVID-19 vaccine/booster?

Very interested	1
Somewhat interested	2
Not very interested	3
Not at all interested	4
I'm not interested in receiving the flu vaccine	5
I'm not interested in receiving the COVID-19 vaccine/booster	6
I'm not interested in receiving either the flu vaccine or COVID-19 vaccine/booster	7
Don't know	98

PN: ASK IF CODE 1 TO 4 SELECTED AT E14

E15. Why did you say that you would be **[INSERT D14 RESPONSE]** in receiving the flu vaccine at the same time as the COVID-19 vaccine?

PN: ASK IF RECEIVED FLU VACCINE THIS SEASON (E2 =1, THIS FLU SEASON)

E16. Did you receive your flu vaccination at the same time as the COVID vaccine/booster this flu season (that is, between September and now).

Yes, I received flu vaccine and COVID-19 vaccine/booster at the same time	1
No, I received flu vaccine and COVID-19 vaccine/booster at different times	2
No, I did not receive COVID-19 vaccine/booster	3
Prefer not to say	99

E17. Why did you **[PN: IF NO AT E16 = 2]** receive influenza vaccination at the same time as the COVID booster this flu season?

SECTION F: FLU VACCINE EXPERIENCE

PN: ASK IF RECEIVED FLU SHOT AT A PHARMACY (E4a = 2)

F1. Earlier you mentioned that you received your last flu vaccine at a **pharmacy**. How did you schedule the flu vaccine shot?

I made an appointment specifically to get the flu vaccine	1
I walked-in to the pharmacy just to get the flu vaccine	2
I made an appointment for the flu vaccine as I was going in for other medications/products	3
I was in the pharmacy for other medications/products so decided to get the flu vaccine, but did not book an appointment for it	4
Other, please specify _____	96
Don't recall	98
Prefer not to say	99

ASK ALL

F2. What motivated you to get your flu vaccine at a **[INSERT E4a RESPONSE]**?

F3. How satisfied were you with your overall experience of receiving a flu vaccine at a **[INSERT E4a RESPONSE]**?

Very satisfied	1
Somewhat satisfied	2
Neither satisfied nor dissatisfied	3
Somewhat dissatisfied	4
Not at all satisfied	5
Don't know/Not sure	98
Prefer not to say	99

PN: ASK IF CODE 1 OR 2 SELECTED AT F3

F4. Why did you say that you are **[INSERT F3 RESPONSE]** with your experience of receiving the flu vaccine at a **[INSERT E4a RESPONSE]**?

SECTION G: UNDERSTANDING NON-PHARMACY FLU VACCINATION RECEIPENTS

PN: ASK THIS SECTION FROM THOSE WHO DID NOT GET FLU VACCINE AT A PHARMACY (NOT E4a=2)

G1. Earlier you mentioned that you did not receive your last flu vaccine at a pharmacy. What are your reasons for not receiving the flu vaccine at a pharmacy? *Please be as descriptive as possible.*

G2. Which of the following factors are a barrier for you in receiving the flu vaccine at a pharmacy? (Select all that apply)

PN: RANDOMIZE

Need to book an appointment to get flu vaccine at a pharmacy	1
Difficulty booking an appointment	2
The vaccine was not offered at my usual/a convenient location	3
Do not feel safe getting flu vaccine at a pharmacy	4
Limited appointment availability	5
Transportation to get to the pharmacy was a problem	6
I didn't know how to schedule an appointment	7
Concern about being exposed to COVID-19	8
Lack of walk-in options	9
I prefer getting the flu vaccine from a/my physician	10
Lack of comfort getting flu vaccine from a pharmacist	11
I believe other health care providers (e.g. physicians, nurses) are more qualified to administer a vaccine	12
Lack of knowledge of flu vaccines among pharmacists	13
Pharmacy offering only flu vaccine, no other vaccinations	14
Not being able to bring in young children or other family members	15
I wasn't aware that flu vaccines are available at pharmacies	99
Other barriers – please specify_____	96

G3. Which of the following factors would motivate you to consider receiving the flu vaccine at a pharmacy? (Select all that apply)

PN: RANDOMIZE

More appointments available	1
Easy to book appointment	2
Extended hours to get flu vaccine at a pharmacy	3
Convenient location	4
Easy transportation to get to the pharmacy	5

Faster / less time consuming to get the vaccine	6
Walk-in appointment available	7
Knowledgeable pharmacists	8
Easier to get the vaccine when visiting the pharmacy for other medication / products	9
Availability of a nurse to give the vaccine at a pharmacy	10
Availability of a physician to give the vaccine at a pharmacy	11
Pharmacy offering all vaccinations, not just flu vaccines	12
Pharmacy offering only flu vaccine, no other vaccinations	13
Recommendation from my family physician	14
Recommendation from a friend / relative	15
Ability to discuss flu vaccine with the pharmacist	16
Other factors – please specify_____	96
Nothing would motivate me to get a flu vaccine at a pharmacy	99

SECTION H: ENHANCED VACCINES

PN: SHOW THIS SECTION TO 65 YEARS OR OLDER RESPONDENTS ONLY

H1. Did you know that there are different influenza vaccines made for adults less than 65 years of age and for people 65 years of age and older?

Yes	1
No	2

PN: SHOW THE BELOW ON A SEPARATE SCREEN AFTER H1
There are, in fact, different influenza vaccines made for adults less than 65 years of age and for people 65 years of age and older. Enhanced influenza vaccines are designed to give older adults better protection against the flu.

H2. Did you know that there is more than one type of enhanced influenza vaccine made specifically for people 65 years of age and older?

Yes	1
No	2

H2b. How familiar are you in general with enhanced influenza vaccines made specifically for people 65 years of age and older?

Very familiar	1
Somewhat familiar	2
Not very familiar	3
Not at all familiar	4
Don’t know/Not sure	98

PN: SHOW THE BELOW ON A SEPARATE SCREEN AFTER H2b
There is, in fact, more than one type of enhanced influenza vaccine specifically formulated and approved for use in for people 65 years of age and older. A higher dose, or the addition of an adjuvant, which is an ingredient that enhances the body’s immune response, can be used.

H3. Have you ever discussed **enhanced influenza vaccines** with any of the following health care providers? *Please select all that apply.*

General Practitioner / Family Physician	1
Another physician	2
Pharmacist	3
Nurse	4
Other, please specify _____	96
I have never discussed enhanced flu vaccines with a healthcare provider	99

H4. Please indicate whether you agree with each of the following statements about enhanced influenza vaccines.

		Strongly disagree	Somewhat disagree	Somewhat agree	Strongly agree	Don't know/Not sure	Prefer not to say
1	Enhanced vaccines help better protect older adults from the seasonal flu	1	2	3	4	98	99
2	It is important that older Canadians have access to enhanced vaccines	1	2	3	4	98	99
3	Enhanced vaccines should be available free of charge to any older Canadian who wants one	1	2	3	4	98	99

H5. How important is it for you to have access to an enhanced influenza vaccine?

Very important	1
Somewhat important	2
Neither important nor unimportant	3
Not very important	4
Not at all important	5
Don't know/Not sure	98
Prefer not to say	99

H6. If your province/territory offered enhanced influenza vaccines, would it make you more likely to get vaccinated against the flu?

Much more likely	1
Somewhat more likely	2
No change in likelihood	3
Somewhat less likely	4
Much less likely	5
Don't know/Not sure	98
Prefer not to say	99

SECTION I: HIGH-RISK GROUPs

I1a. Before today, were you aware that the National Advisory Committee on Immunization (NACI) has identified certain groups as being high risk of influenza-related complications or hospitalization, for whom influenza vaccine is particularly recommended?

Yes	1
No	2

PN: ASK IF YES AT I1a

I1b. How knowledgeable would you say you are about the recommendation from the National Advisory Committee on Immunization (NACI) regarding influenza vaccine for certain group of individuals identified as high-risk group?

Very knowledgeable	1
Somewhat knowledgeable	2
Not very knowledgeable	3
Not at all knowledgeable	4
Don’t know/Not sure	98

I2. As far as you know, do you belong to a high-risk group for whom influenza vaccine is particularly recommended?

Yes	1
No	2
I’m not sure	99

I3. Knowing that there are certain groups of people considered high-risk for influenza-related complications or hospitalization who NACI strongly recommends receives a flu vaccine in particular, how important do you feel it is for these individuals to get the flu vaccine?

Very important	1
Somewhat important	2
Neither important nor unimportant	3
Not very important	4
Not at all important	5
Don’t know/Not sure	98
Prefer not to say	99

PN: ASK IF CODE 1, 2, 3, 4 OR 5 AT I3

I3b. Why do you say it is **[INSERT I3 RESPONSE]** for high-risk group individuals to get the flu vaccine?

SECTION J: DEMOGRAPHICS

J1. Please provide the first half of your postal code (e.g. K1K). [Open-end]

_____ (A2A)

J2. People living in Canada come from many different cultural and racial backgrounds. The following question will help us to better understand the experiences of the communities that we serve. Do you consider yourself to be . . . (Select all that apply)

First Nation	1
Inuit	2
Métis	3
Indigenous/Aboriginal (not included above)	4
Arab	5
Black (North American, Caribbean, African, etc.)	6
Chinese	7
Filipino	8
Japanese	9
Korean	10
Latin American	11
South Asian (East Indian, Pakistani, Sri Lankan etc.)	12
Southeast Asian (Vietnamese, Cambodian, Malaysian, Laotian, etc.)	13
West Asian (Iranian, Afghan, etc.)	14
White (North American, European, etc.)	15
Other (specify): ________________________	96
Prefer not to say	99

J3. What is your current marital status?

Single / never married	1
Married	2
Common law	3
Separated	4
Divorced	5
Widowed	6
Prefer not to say	99

J4. Including yourself, how many people live in your household, counting adults and children?

_____ # people

- One person (myself)
- Prefer not to say

PN: ASK IF >1 AT J4

J5. How many people in each of the following age groups (if any) do you have living with you in your household?

<18 years old	___#
18-34 years old	___#
35-44 years old	___#
45-54 years old	___#
55-44 years old	___#
65 years of age or older	___#
Prefer not to say	99

SUM=J4

J6. What is the highest level of education you have completed?

Less than high school	1
High school	2
Some college or university	3
College graduate or CEGEP	4
Bachelor's degree	5
Master's or professional degree	6
Doctorate	7
Prefer not to say	99

J7. What is your current employment status?

Work for an employer full-time	1
Work for an employer part time	2
Self-employed	3
Unemployed	4
Student	5
Homemaker	6
Retired	7
Other (please specify) _____	96
Prefer not to say	99

J8. Which of the following best describes your current health insurance coverage?

Public / provincial coverage	1
Private insurance	2
No coverage	3
Don’t know/Not sure	98
Prefer not to say	99

J9. What is your annual household income (from all sources before taxes)?

Less than $20,000	1
$20,000-$39,999	2
$40,000-$69,999	3
$70,000-$99,999	4
$100,000-$119,999	5
$120,000 or more	6
Prefer not to say	99

J10. Which of the following location descriptions best defines where you live? (Select one only)

Rural (population of less than 50,000)	1
Small town (population between 50,000 – 250,000)	2
Large city (population from 250,000 – 1 million)	3
Metropolitan (population of 1 million or more)	4
Don’t know/Not sure	5
Prefer not to say	6

J11. Were you born in Canada?

Yes	1
No	2
Prefer not to say	99

J12. On a scale of one to five, with one being poor and five being excellent, how would you rate your health?

One (poor)	1
Two (fair)	2
Three (good)	3
Four (very good)	4
Five (excellent)	5
Don’t know/Not sure	6
Prefer not to say	99

PN: ASK IF NO AT J12

J13. In what year did you move to Canada?

Record year: XXXX

- o Don't know/Not sure
- o Prefer not to say

PN: ASK IF NO AT J12

J14. In which country were you born?

Afghanistan	1
Algeria	2
Bangladesh	3
Belgium	4
China	5
Colombia	6
France	7
Germany	8
Greece	9
Guyana	10
Hong Kong	11
India	12
Iran	13
Italy	14
Jamaica	15
Korea, Republic of (South Korea)	16
Lebanon	17
Netherlands	18
Pakistan	19
Philippines	20
Poland	21
Portugal	22
Romania	23
Russia	24
Sri Lanka	25
Taiwan	26
Trinidad and Tobago	27
Turkey	28
Ukraine	29
United Kingdom	30
United States	31
Vietnam	32
Other (please specify) ______	96
Don't know/Not sure	98
Prefer not to say	99

J15. Are you currently suffering from or being treated for any of the following conditions? *Please select all that apply.*

Asthma	1
Another chronic lung disease such as emphysema, chronic bronchitis or cystic fibrosis	2
A heart condition such as coronary heart disease, heart failure, heart attack	3
High blood pressure	4
Cancer	5
Diabetes or other metabolic diseases	6
Chronic liver disease	7
Chronic kidney disease	8
Immune disorder or immune suppression such as chemotherapy, radiation, steroid use or an organ transplant	9
Spleen problems or removal	10
Anemia / thalassemia / hemoglobinopathy	11
Morbid obesity (BMI > 40)	12
Conditions that compromise management of respiratory secretions, with increased risk of aspiration	13
Chronic cerebrospinal fluid (CSF) leak	14
None of the above	98
Don’t know	99

J16. The National Advisory Committee on Immunization (NACI) has identified certain groups as high-risk group for whom influenza vaccine is particularly recommended. Do you identify with any of these? *Please select all that apply.*

Pregnant individuals	1
Residents of nursing homes and other long-term care facilities	2
None of the above	99

Thank you for participating in the survey. This brings us to the end of the survey.
